# Tracking Down Antibiotic-Resistant *Pseudomonas aeruginosa* Isolates in a Wastewater Network

**DOI:** 10.1371/journal.pone.0049300

**Published:** 2012-12-19

**Authors:** Céline Slekovec, Julie Plantin, Pascal Cholley, Michelle Thouverez, Daniel Talon, Xavier Bertrand, Didier Hocquet

**Affiliations:** 1 Service d'Hygiène Hospitalière, Centre Hospitalier Universitaire, Besançon, France; 2 Unité Mixte de Recherche 6249 Chrono-environnement, Université de Franche-Comté, Besançon, France; Universitätsklinikum Hamburg-Eppendorf, Germany

## Abstract

The *Pseudomonas aeruginosa*-containing wastewater released by hospitals is treated by wastewater treatment plants (WWTPs), generating sludge, which is used as a fertilizer, and effluent, which is discharged into rivers. We evaluated the risk of dissemination of antibiotic-resistant *P. aeruginosa* (AR-*PA*) from the hospital to the environment via the wastewater network. Over a 10-week period, we sampled weekly 11 points (hospital and urban wastewater, untreated and treated water, sludge) of the wastewater network and the river upstream and downstream of the WWTP of a city in eastern France. We quantified the *P. aeruginosa* load by colony counting. We determined the susceptibility to 16 antibiotics of 225 isolates, which we sorted into three categories (wild-type, antibiotic-resistant and multidrug-resistant). Extended-spectrum β-lactamases (ESBLs) and metallo-β-lactamases (MBLs) were identified by gene sequencing. All non-wild-type isolates (*n* = 56) and a similar number of wild-type isolates (*n* = 54) were genotyped by pulsed-field gel electrophoresis and multilocus sequence typing. Almost all the samples (105/110, 95.5%) contained *P. aeruginosa*, with high loads in hospital wastewater and sludge (≥3×10^6^ CFU/l or/kg). Most of the multidrug-resistant isolates belonged to ST235, CC111 and ST395. They were found in hospital wastewater and some produced ESBLs such as PER-1 and MBLs such as IMP-29. The WWTP greatly reduced *P. aeruginosa* counts in effluent, but the *P. aeruginosa* load in the river was nonetheless higher downstream than upstream from the WWTP. We conclude that the antibiotic-resistant *P. aeruginosa* released by hospitals is found in the water downstream from the WWTP and in sludge, constituting a potential risk of environmental contamination.

## Introduction

*Pseudomonas aeruginosa* is a rod-shaped Gram-negative aerobic bacterium that can grow in many niches, but prefers moist environments. It is found in both low-nutrient or oligotrophic environments and highly nutritious environments, such as sewage and the human body.

*P. aeruginosa* is a common hospital-acquired pathogen of the respiratory and urinary tracts, in all hospital departments, but particularly intensive care units, in which 15% of healthcare-associated infections are attributed to this pathogen [1]. Hospital outbreaks linked to multidrug-resistant strains of *P. aeruginosa* are widely reported [2]–[5]. The intrinsic resistance of *P. aeruginosa* to many classes of antibiotics and its capacity to acquire resistance to almost all effective antibiotics during treatment render infections with this microorganism very difficult to treat [6]–[8]. Multidrug-resistant *P. aeruginosa* is thought to emerge principally at hospitals, where large amounts of antibiotics are used [9]. Antibiotic resistance in *P. aeruginosa* mostly results from chromosomal mutations, but may also be acquired by horizontal gene transfer [10]. Resistance to β-lactams is of particular concern in clinical practice. High-level resistance to these compounds is achieved by AmpC cephalosporinase overproduction or by the production of acquired β-lactamases with an extended spectrum (*i.e.* ESBLs, MBLs and extended-spectrum oxacillinases) [11].

*P. aeruginosa* also causes community-acquired infections, including folliculitis and ear infections acquired by recreational exposure to water containing the bacterium and keratitis, particularly in patients who wear contact lenses. Although some *P. aeruginosa* strains are shared by cystic fibrosis patients, the source of the contamination remains unknown and could include the natural environment (*e.g.* soil and water) as reservoir.

Typically, the wastewater from urban sewerage systems (containing rainwater, hospital and urban wastewater) is treated at a wastewater treatment plant (WWTP), to generate clean effluent for discharge into rivers and of sludge, which may be used as a fertilizer. Antibiotic-resistant *P. aeruginosa* (AR-*PA*) strains are found in the effluent of WWTPs and in the river water downstream from these plants [12]. However, the source of these resistant bacteria has yet to be clearly established. The potential dissemination of *P. aeruginosa* from hospitals to natural environments may contribute an increase in the number of community-acquired infections with multidrug-resistant pathogens.

We evaluated the risk of AR-*PA* dissemination from hospitals to the environment. We quantified the *P. aeruginosa* load throughout the wastewater network and determined the antibiotic resistance profile of the isolates obtained, focusing in particular on enzyme-based mechanisms of resistance to β-lactams. We then determined the genotype of antibiotic-resistant isolates, to facilitate the tracking of their spread from the hospital to the environment. We conclude that the AR-*PA* strains released by hospitals are found in the water downstream from the WWTP and in sludge, constituting a potential risk of environmental contamination.

## Materials and Methods

### Study setting

**Figure 1:**
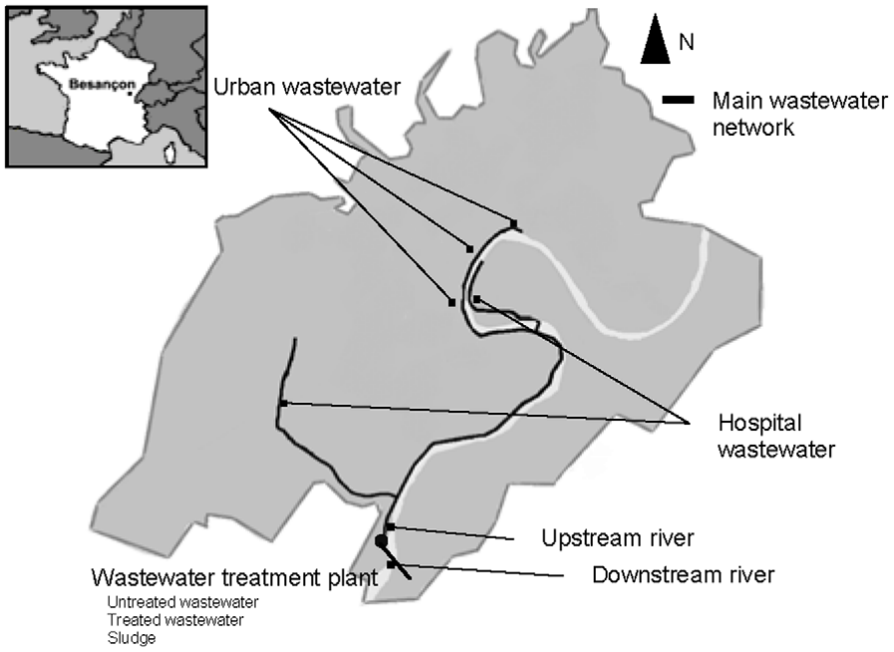
Map of the study area. The large map indicates the precise location of the sampling sites, with the inset map indicating the location of the area in France.

This study was carried out in the city of Besançon, in eastern France (130,000 inhabitants). The WWTP studied serves approximately 120,000 people and had a mean hydraulic load in 2011 of 30,000 m^3^ per day. The effluent treated by the plant includes effluents from two University hospital sites, with 800 and 400 beds, urban wastewater and rainwater (Figure 1). The water is treated by a sequence of three typical treatments (sedimentation, biological content degradation and effluent polishing) before sludge production and the discharge of the treated effluent into the river. Of the 7,500 metric tons of sludge produced each year, 4,500 metric tons are used as fertilizer. The river upstream from the WWTP contained treated water originating from medical facilities 80 km upstream from the city of Besançon. Mean monthly rainfall was 46 mm during the study period, and 88 mm over the last decade.

### Wastewater sampling

Samples were collected from 11 sites distributed throughout the wastewater network of the city (Figure 1). Each collecting point was sampled weekly, over a 10-week period, between January and April 2011. We collected (*i*) wastewater from the two sites of our hospital (containing only hospital effluent and rainwater), (*ii*) three urban wastewater samples independent of hospitals and healthcare facilities. We also collected samples within the WWTP: (*iii*) untreated water containing urban and hospital wastewater (*n* = 1), (*iv*) treated water before its discharge into the river (*n* = 2; the daily samples consisted of pools of aliquots taken from each 10 m^3^ volume of water) and (*v*) the anaerobically digested sludge ready for spreading on farm fields (*n* = 1). We also collected samples of river water (*vi*) upstream and (*vii*) downstream from the WWTP.

### *P. aeruginosa* load determination

Samples were analyzed within eight hours of collection. We quantified *P. aeruginosa* in heavily loaded samples (urban wastewater, untreated water, sludge and hospital wastewater), by serial dilution method, after appropriate dilution in sterile water. A 100-µl aliquot of each diluted sample was plated on a *Pseudomonas*-specific agar plate (CN/agar, Bio-Rad, Marnes-La-Coquette, France). The *P. aeruginosa* load of lightly contaminated samples (treated water and river water) was assessed by the membrane filtration method. A 100-ml aliquot of the water to be tested was passed through a filter with 0.45-µm pores, which was then placed on a CN/agar plate. CN/agar plates were incubated for 48 h at 37°C. *P. aeruginosa* colonies were initially detected by standard microbiology methods (*i.e.* colony morphology, positive oxidase reaction, pigment production). Identification was then confirmed with a biochemical test (ID 32 GN, Biomérieux, Mercy l'étoile, France). We analyzed a maximum of five colonies per plate, selected on the basis of colony morphology, and these colonies were stored in brain heart infusion broth supplemented with 20% glycerol at −80°C until analysis.

### Bacterial clearance rates

The clearance rate at the WWTP was determined as follows:
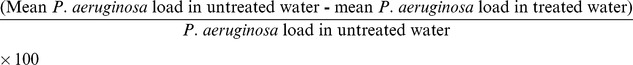



### Antimicrobial susceptibility testing and β-lactamase identification

We aimed to assess the diversity of the resistance profiles within each sample. We used colony morphology methods, despite the limitations of this method (maximum of five morphotypes per sample. We assessed the activity of 16 antibiotics from four different classes (non-carbapenem β-lactams: cefepime, piperacillin, piperacillin-tazobactam, cefotaxime, ticarcillin, ticarcillin-clavulanate, ceftazidime, aztreonam; carbapenems: meropenem, imipenem; aminoglycosides: gentamicin, tobramycin, amikacin; fluoroquinolones: ciprofloxacin) against the selected *P. aeruginosa* isolates by the disk diffusion method, as recommended by the Antibiogram Committee of the French Society for Microbiology [13]. Three resistance phenotypes were defined: ‘wild-type’ (susceptible to all the tested antibiotics), ‘resistant’ (not susceptible to antibiotics from one or two classes), and ‘multidrug-resistant’ (not susceptible to antibiotics from three or more classes). We also identified ESBLs and MBLs in isolates resistant to third-generation cephalosporins, by the phenotypic method described elsewhere [14]. For isolates considered positive by this approach, the enzymes involved were identified by PCR and sequencing with primers targeting ESBL- and MBL-encoding genes [15].

### Genotyping by pulsed-field gel electrophoresis (PFGE)

The clonality of strains was investigated by PFGE, with *Dra*I digestion, as previously described [16]. The electrophoresis was performed at 14°C and 5.5 V/cm using the CHEF DR III apparatus (Bio-Rad Laboratories, Hercules, Calif.) in 15-cm by 20-cm 1% agarose gels (Life Technologies, Saint Aubin, France). We used two successive sequences of electrophoresis. The first sequence lasted 12 h with a constant switch time of 20 s. The second sequence lasted 17 h with a switch time increasing from 5 s to 15 s. Samples of *Sma*I-restricted DNA from *Staphylococcus aureus* NCTC 8325 were included in each run as an internal reference. The banding patterns were analyzed by scanning photographic negatives. GelCompar software was used for cluster analysis (Applied Maths, Kortrijk, Belgium). Each strain was first compared with all other strains. The Dice correlation coefficients were grouped and the UPGMA clustering algorithm was used to depict the groups as a dendrogram. Pulsotypes (PTs) were defined according to international recommendations [17].

### Genotyping by multi-locus sequence typing (MLST)

MLST was performed according to the protocol of Curran *et al.* [18], as modified by van Mansfeld *et al.* [19]. Nucleotide sequences were determined for internal fragments of the *acsA*, *aroE*, *guaA*, *mutL*, *nuoD*, *ppsA* and *trpE* genes, on both strands, and were compared with sequences in the *P. aeruginosa* MLST website (http://pubmlst.org/paeruginosa/) for the assignment of allele numbers and sequence types (ST).

### Analysis of MLST data

We investigated the evolutionary relationship between isolates with the minimal spanning tree (MST) algorithm, which is based on allelic profiles. The MST algorithm is a graphical tool that links the nodes by unique minimal paths in a given dataset, *i.e.* the total summed distance of all the branches is minimized [20]. The algorithm uses the ST with the highest number of single locus variants (SLVs) as a root node, from which it derives the other STs. Using a stringent definition of 5/7 shared alleles, MST then connects all the strains and links all related STs into clonal complexes. Singletons were thus defined as STs with at least three allelic mismatches with all other STs.

#### Ethics statement

This study was approved by the ‘Comité d'Etude Clinique’ ethics committee of Besançon Hospital, Besançon, France. All the water and sludge samples came from public areas and facilities. All necessary permits were obtained by Christian IMPERAS, Technical Director of Water Supply and Water Treatment Service of the City of Besançon. We confirm that the location is not privately-owned or protected in any way. We confirm that the field studies did not involve endangered or protected species.

### Statistical analysis

Continuous variables were compared in non-parametric Kruskal-Wallis tests and categorical variables were compared in Pearson's Chi-squared test. All tests were two-tailed, and a *p*-value of less than 0.05 was considered statistically significant.

## Results

### *P. aeruginosa* load of the samples

**Figure 2:**
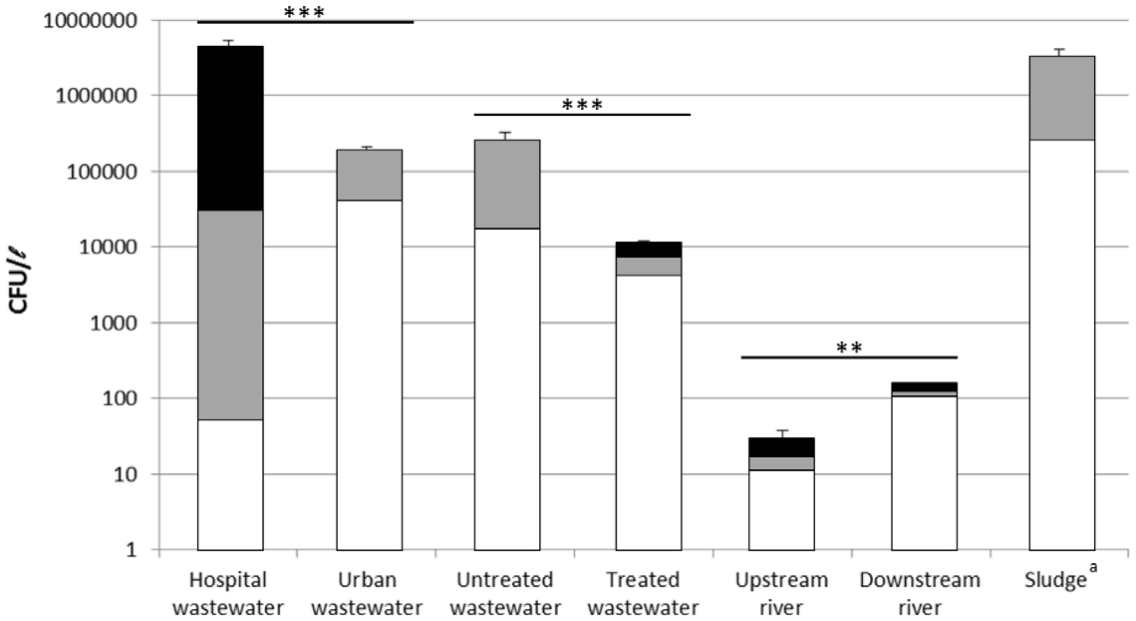
*P. aeruginosa* load of the water and sludge at the various sampling points. The proportion of the isolates with each resistance profile (white: wild-type; gray: resistant; black: multidrug-resistant) is shown. *^a^* The bacterial load in the sludge is expressed in CFU per kg.

Over the study period, we processed 110 samples, 105 (95.5%) of which tested positive for *P. aeruginosa*. The *P. aeruginosa* load at the sampling locations is detailed in Figure 2. The *P. aeruginosa* load of hospital wastewater was 25 times higher than that of urban wastewater (4.46×10^6^ *vs.* 0.18×10^6^ CFU/l, respectively; *p* = 0.0001). The effluent treatment carried out by the WWTP resulted in an overall *P. aeruginosa* clearance rate of 94.0% (2.5×10^5^ CFU/l in untreated water *versus* 1.1×10^4^ CFU/ml in treated water; *p* = 0.0001). However, wastewater treatment concentrated *P. aeruginosa* in the sludge, resulting in a high load (2.95×10^6^ CFU/kg). The *P. aeruginosa* load in the river downstream from the WWTP was significantly higher than that in the river upstream from the WWTP (128 *versus* 27 CFU/l, respectively, *p* = 0.0012).

### Resistance profile analysis

**Figure 3:**
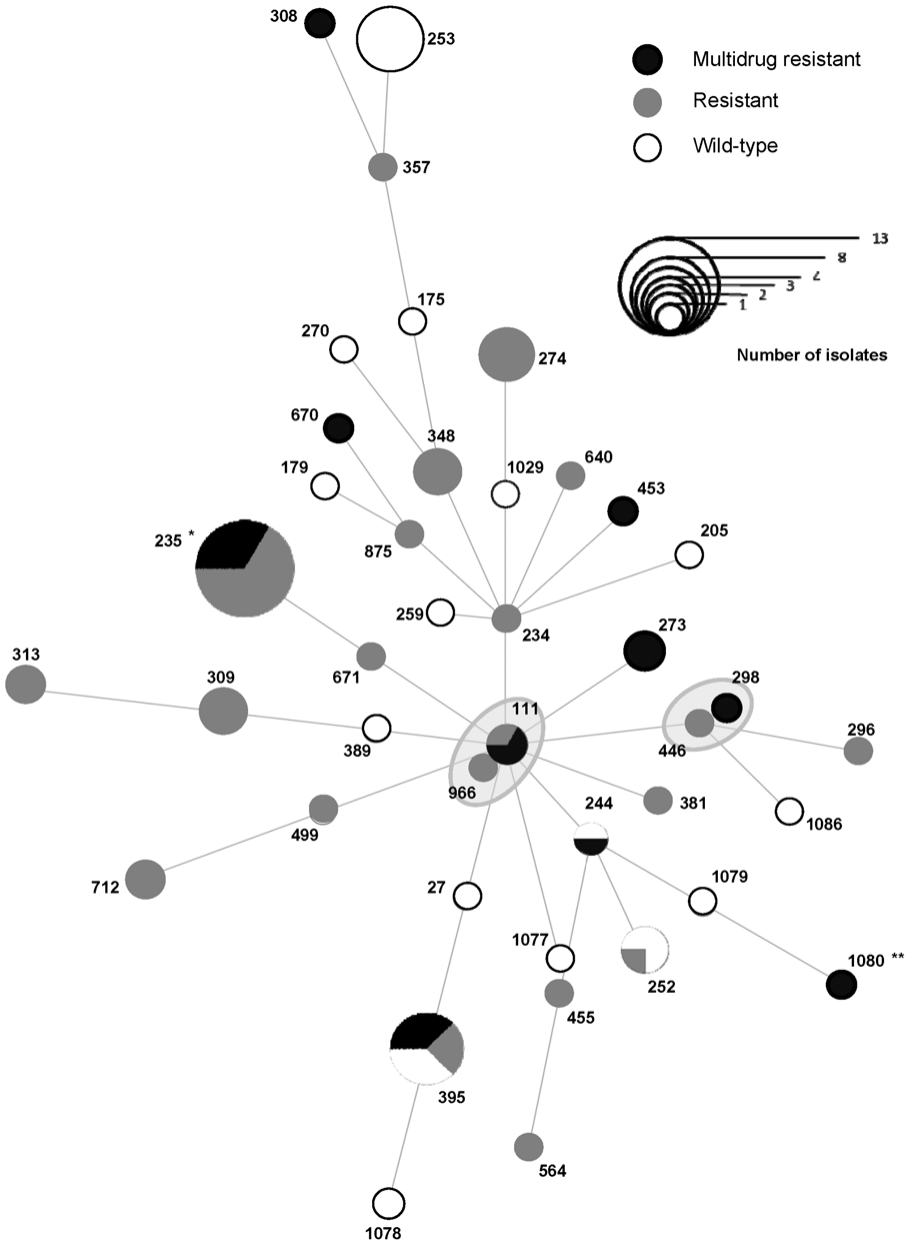
Minimal spanning tree analysis of *P. aeruginosa* strains based on MLST data and resistance profiles. The proportion of isolates with each antibiotic resistance profile is indicated for each ST. Each dot represents a single ST, with a diameter proportional to the number of isolates (see frame). The relationships between strains are indicated by the connections between the dots and the lengths of the branches linking them. The gray areas surround STs belonging to the same clonal complex. * One ST235 isolate (from hospital wastewater) produced the ESBL PER-1 and another (from the water treated by the WWTP) produced the MBL IMP-29. **: One ST1080 isolate (from hospital wastewater) produced PER-1.

Based on colony morphology (see above), we selected 238 isolates for further testing: 38 were isolated from hospital wastewater, 70 from urban wastewater, 88 from the WWTP (27 from untreated water, 49 from treated water and 12 from sludge) and 42 were isolated from the river (13 upstream from the WWTP, 29 downstream from the WWTP). We classified 182 of these 238 isolates as wild-type, 39 as resistant and 17 as multidrug-resistant. Wild-type and resistant strains were ubiquitous, whereas multidrug-resistant strains were found only in hospital wastewater, treated water and the river. For identification of the ESBLs and MBLs harbored by *P. aeruginosa* in the wastewater network, we characterized the enzymatic mechanism of resistance to β-lactams in the 11 isolates resistant to third-generation cephalosporins (cefepime and/or ceftazidime). Eight of these isolates overproduced the chromosomally encoded cephalosporinase AmpC as the sole enzyme conferring resistance to β-lactams. Two *P. aeruginosa* isolates from hospital wastewater produced the ESBL PER-1. One isolate from treated water within the WWTP produced the MBL IMP-29 (Figure 3).

### Genotyping

We determined the PFGE profile of all 56 non-wild-type isolates (39 resistant and 17 multidrug-resistant isolates) and a similar number (54 of the 182) of wild-type isolates selected at random. Of the 110 *P. aeruginosa* isolates analyzed, 32 were from hospital wastewater, 26 were from urban wastewater, 36 were from the WWTP and 16 were from the river (see details in the Table S1). This analysis yielded 80 different PTs, 65 of which were unique (Table S1). Fifteen multiple PFGE patterns were each found in two to eight strains: nine PTs included two strains, two PTs included three strains, two PTs included four strains, one PT included five strains and one PT included eight strains.

**Figure 4:**
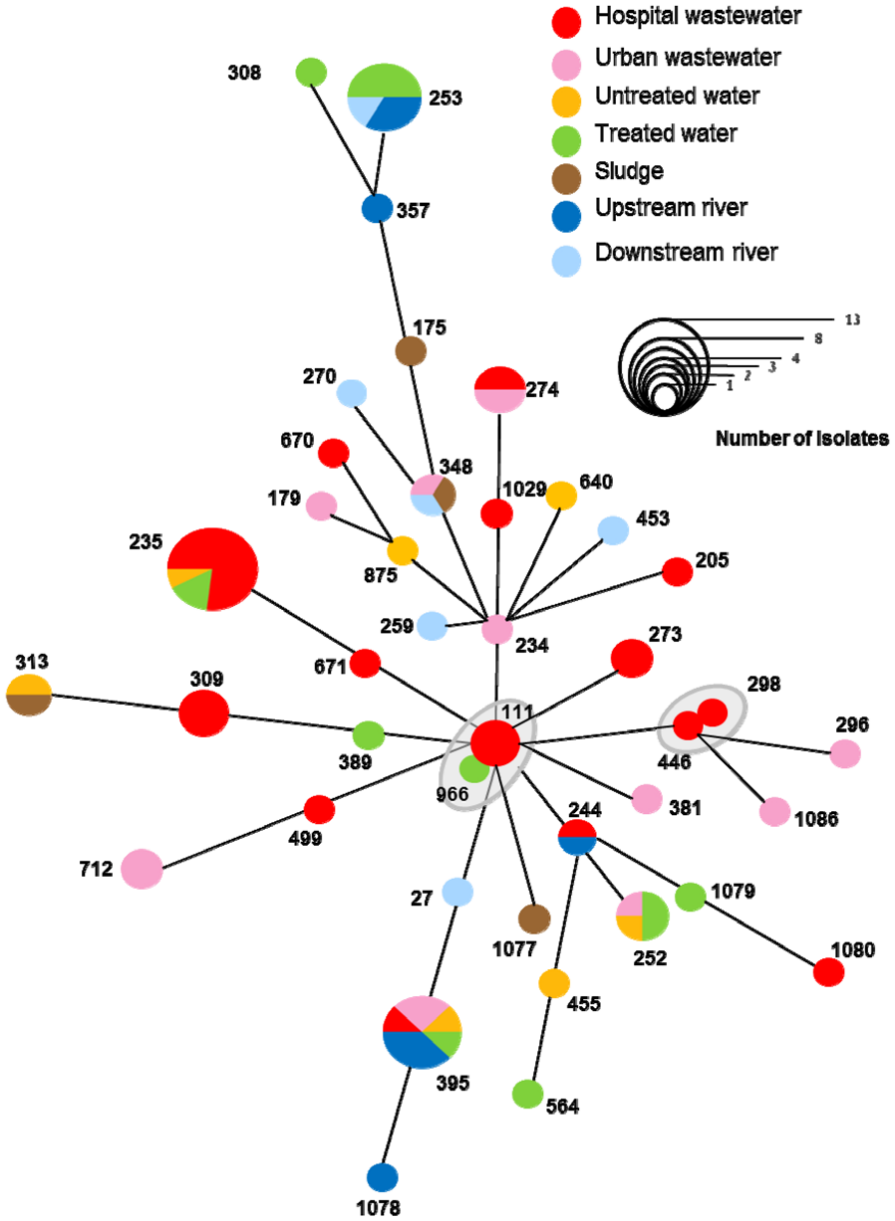
Minimal spanning tree analysis of *P. aeruginosa* strains based on MLST data and isolation sites.

As PFGE typing is more discriminatory than MLST, we assumed that all the isolates with a given PT belonged to the same ST. We therefore determined the MLST profiles of the 54 PTs including at least one resistant or multi-resistant isolate. These 54 PTs included 81 isolates: 25 wild-type and all 56 resistant or multidrug-resistant isolates. These PTs belonged to 41 different STs. Three major STs were identified: ST235 (including 13 isolates from hospital wastewater and WWTP); ST395 (including eight isolates from the various sampling points of the wastewater network) and ST253 (including six isolates from the river and the WWTP) (Figure 4). We determined the clonal relationship between STs by the MST method based on allelic profile [21]. Two allelic mismatches were allowed for group definition, as in group definition with eBURST. We represented the MST of the isolates with respect to their resistance profiles (Figure 3) or their origins (Figure 4). The 41 STs identified were distributed into 35 singletons (73 isolates) and two clonal complexes of six isolates (CC111, which included ST111 and ST966; and CC446, which included ST446 and ST296). The details of the STs (antibiotic resistance profile and origin) are provided in Table S1.

## Discussion

### *P. aeruginosa* in the wastewater network

More than 95% of the water samples tested positive for *P. aeruginosa*, demonstrating the presence of this species throughout the wastewater network (Figure 2). The clearance rate at the WWTP was high (94%; Figure 2), but we nevertheless found resistant and multidrug-resistant isolates in the treated water before its release in the river and in the sludge produced by the WWTP. Of particular interest, the treated water was found to contain an isolate producing the MBL IMP-29 recently described at our hospital [22]. The PT of the clinical isolates described by Jeannot *et al.* differed from that of the isolate in our series (data not shown), suggesting horizontal transfer of the resistance gene. Such resistance transfer has clearly been described in WWTPs, in the absence of antibiotic selection [23]. We found an unexpectedly high concentration of *P. aeruginosa* in the sludge produced by the WWTP (2.95×10^6^ CFU/kg), a concentration within the range generally found in hospital wastewater (Figure 2). This sludge is used as a fertilizer and is applied to the soil directly, without dilution. Thus, treated water and sewage sludge may constitute a potential risk of environmental contamination with AR-*PA*.

### *P. aeruginosa* release from the hospital

The release of AR-*PA* into the environment by hospitals is a controversial issue. *P. aeruginosa* isolates resistant to ciprofloxacin or producing VIM-type MBLs have been obtained from wastewater and hospital discharge sites [12], [24], [25]. By contrast, a pilot study showed that our hospital did not release AR-*PA* into the environment [26]. However, hospital wastewater is a highly selective environment that may contribute to the maintenance of resistant bacteria discharged into the natural environment [27], [28]. This study was designed to address these issues. The *P. aeruginosa* load was found to be significantly higher in hospital wastewater than in urban wastewater, consistent with previous findings [12]. This is particularly important given the much higher dilution phenomenon in hospitals than in the community (1,000 l of wastewater per bed per day versus only 150 l per inhabitant per day in the community). As expected, the STs found in hospital effluent (ST111, ST235, ST395 and ST446) had all previously been isolated from patients hospitalized in our medical facility [11]. We have shown that most of the multidrug-resistant isolates from hospitals belong to a few clonal types [11]. We therefore hypothesized that, in this series, the high frequency of resistance to antibiotics was due to the overrepresentation of certain resistant clones. Resistant isolates from hospital wastewater were genotyped and most were found to belong to ST235, ST395, ST309, ST273, CC111 and CC446 (Table S1). The overall frequency of resistant *P. aeruginosa* isolates was higher in hospital wastewater than among isolates obtained directly from clinical samples (Bertrand X., personal data). This suggests that the hospital wastewater environment (containing antibiotics, disinfectants and heavy metals, in particular) [29] favors these antibiotic-resistant clones over susceptible clones.

### Tracking down antibiotic-resistant *P. aeruginosa* from the hospital

We assessed the fate of AR-*PA* clones released into the wastewater network by the hospital, including their presence in the WWTP, downstream river water and sludge. Genotyping of the resistant isolates throughout the network showed that three STs or clonal complexes present in the hospital wastewater (ST235, ST395, and CC111) were also present in the WWTP or the river. ST235 was represented by 13 resistant and multidrug-resistant strains, mostly isolated from hospital wastewater (*n* = 10), but also found in untreated water (*n* = 1) and in treated water released from the WWTP (*n* = 2). Two ST235 isolates produced either the MBL IMP-29 (see above) or the ESBL PER-1. ST235 clones producing PER-1 have spread worldwide and have also been identified at our hospital [11]. CC111 includes isolates from ST111 and ST966 (Figures 3 and 4). Resistant CC111 isolates were isolated from both hospital wastewater (*n* = 3) and from treated water from the WWTP (*n* = 1). Finally, we found eight ST395 isolates at various points in the network: in hospital (*n* = 1) and urban (*n* = 2) wastewater, in untreated water from the WWTP (*n* = 2) and in the river downstream from the WWTP (*n* = 3). We previously showed that the widespread ST235, ST111 and ST395 were overrepresented among clinical AR-*PA* isolates in eastern France [11]. Moreover, a major outbreak involving more than 200 patients in our hospital between 1997 and 2009 was due to a ST395 clone with variable antibiotic resistance, as described in a previous study [30]. We therefore assume that the antibiotic-resistant isolates from ST235, CC111 and ST395 were probably released into the wastewater network by the hospital.

Surprisingly, we retrieved an unexpectedly high proportion of multidrug-resistant *P. aeruginosa* isolates from the river upstream from the WWTP (Figure 2). As hospitals are a major source of multidrug-resistant strains, we speculate that these multidrug-resistant *P. aeruginosa* probably came from the discharge, along the length of the river, from other plants treating wastewater from medical facilities. For example, there is a 1200-bed hospital located 80 km upstream. These data suggest that antibiotic-resistant bacteria may be maintained in the river water. Other studies have found traces of antibiotics (*e.g.* amoxicillin, sulfamethoxazole and fluoroquinolones) in French rivers [31], [32] that might contribute to the maintenance of resistant population in this environment [33].

### Conclusion

We show here that *P. aeruginosa* is ubiquitous in the water network and that antibiotic-resistant (and, in particular, multidrug-resistant) strains are released from hospitals. Water treatment is effective at removing *P. aeruginosa* from the effluent, but does not decrease the proportion of antibiotic-resistant strains. Treated water and sewage sludge may therefore constitute a risk of environmental contamination with AR-*PA* released by hospitals.

## Supporting Information

Table S1 **Sequence type, PFGE pattern, resistance profile and site of isolation of the** ***P. aeruginosa*** **isolates.**
(DOC)
