# Supplementary material for: Tracking Down Antibiotic-Resistant Pseudomonas aeruginosa Isolates in a Wastewater Network
Source: PLoS One. 2012 Dec 19;7(12):e49300. doi: 10.1371/journal.pone.0049300 (PMC3526604; doi:10.1371/journal.pone.0049300)
Supplement: Table S1 — Sequence type, PFGE pattern, resistance profile and site of isolation of the P. aeruginosa isolates. (DOC) [file pone.0049300.s001.doc]

**Table S1:** Sequence type (ST), PFGE pattern, resistance profile and site of isolation of *P. aeruginosa* isolates from the Besançon wastewater network.

| **ST** | **PFGE pattern** | **Resistance profile*^a^*** | **No. of isolates** | **Sampling location** |
| --- | --- | --- | --- | --- |
| **27** | **1** | wild-type | 1 | downstream river |
| **111** | **22** | multidrug-resistant | 2 | hospital wastewater |
| **111** | **28** | resistant | 1 | hospital wastewater |
| **175** | **47** | wild-type | 1 | sludge |
| **179** | **3** | wild-type | 1 | urban wastewater |
| **205** | **80** | wild-type | 1 | hospital wastewater |
| **234** | **27** | resistant | 1 | urban wastewater |
| **235** | **48** | multidrug-resistant | 1 | hospital wastewater |
| **235** | **49** | multidrug-resistant | 1 | hospital wastewater |
| **235** | **49** | multidrug-resistant | 1 | treated wastewater |
| **235** | **49** | resistant | 1 | untreated wastewater |
| **235** | **49** | resistant | 4 | hospital wastewater |
| **235** | **49** | resistant | 1 | treated wastewater |
| **235** | **50** | multidrug-resistant | 1 | hospital wastewater |
| **235** | **50** | resistant | 3 | hospital wastewater |
| **244** | **14** | wild-type | 1 | upstream river |
| **244** | **60** | multidrug-resistant | 1 | hospital wastewater |
| **252** | **31** | wild-type | 1 | urban wastewater |
| **252** | **31** | wild-type | 2 | treated wastewater |
| **252** | **32** | resistant | 1 | untreated wastewater |
| **253** | **7** | wild-type | 1 | treated wastewater |
| **253** | **34** | wild-type | 2 | upstream river |
| **253** | **34** | wild-type | 1 | downstream river |
| **253** | **34** | wild-type | 2 | treated wastewater |
| **259** | **41** | wild-type | 1 | downstream river |
| **270** | **29** | wild-type | 1 | downstream river |
| **273** | **61** | multidrug-resistant | 2 | hospital wastewater |
| **274** | **42** | resistant | 2 | urban wastewater |
| **274** | **58** | resistant | 2 | hospital wastewater |
| **296** | **36** | resistant | 1 | urban wastewater |
| **298** | **55** | multidrug-resistant | 1 | hospital wastewater |
| **308** | **26** | multidrug-resistant | 1 | treated wastewater |
| **309** | **51** | resistant | 1 | hospital wastewater |
| **309** | **72** | resistant | 1 | hospital wastewater |
| **309** | **73** | resistant | 1 | hospital wastewater |
| **313** | **70** | resistant | 1 | sludge |
| **313** | **70** | resistant | 1 | untreated wastewater |
| **348** | **24** | resistant | 1 | urban wastewater |
| **348** | **75** | resistant | 1 | downstream river |
| **348** | **75** | resistant | 1 | sludge |
| **357** | **57** | resistant | 1 | upstream river |
| **381** | **64** | resistant | 1 | urban wastewater |
| **389** | **67** | wild-type | 1 | treated wastewater |
| **395** | **6** | wild-type | 1 | untreated wastewater |
| **395** | **6** | wild-type | 1 | urban wastewater |
| **395** | **20** | multidrug-resistant | 2 | upstream river |
| **395** | **20** | multidrug-resistant | 1 | hospital wastewater |
| **395** | **20** | resistant | 1 | upstream river |
| **395** | **43** | resistant | 1 | urban wastewater |
| **395** | **68** | wild-type | 1 | treated wastewater |
| **446** | **56** | resistant | 1 | hospital wastewater |
| **453** | **54** | multidrug-resistant | 1 | downstream river |
| **455** | **59** | resistant | 1 | untreated wastewater |
| **499** | **10** | resistant | 1 | hospital wastewater |
| **564** | **40** | resistant | 1 | treated wastewater |
| **640** | **21** | resistant | 1 | untreated wastewater |
| **670** | **71** | multidrug-resistant | 1 | hospital wastewater |
| **671** | **39** | resistant | 1 | hospital wastewater |
| **712** | **19** | resistant | 2 | urban wastewater |
| **875** | **23** | resistant | 1 | untreated wastewater |
| **966** | **77** | resistant | 1 | treated wastewater |
| **1029** | **62** | wild-type | 1 | hospital wastewater |
| **1077** | **17** | wild-type | 1 | sludge |
| **1078** | **76** | wild-type | 1 | upstream river |
| **1079** | **13** | wild-type | 1 | treated wastewater |
| **1080** | **4** | multidrug-resistant | 1 | hospital wastewater |
| **1086** | **45** | wild-type | 1 | urban wastewater |

*^a^*Wild-type isolates were susceptible to all the antibiotics tested, resistant isolates were not susceptible to the antibiotics of one or two classes and multidrug-resistant isolates were not susceptible to antibiotics from three or more classes.
